# Lipid Metabolic Alterations in KRAS Mutant Tumors: Unmasking New Vulnerabilities for Cancer Therapy

**DOI:** 10.3390/ijms24021793

**Published:** 2023-01-16

**Authors:** Maria Saliakoura, Georgia Konstantinidou

**Affiliations:** Institute of Pharmacology, Inselspital, INO-F, University of Bern, 3010 Bern, Switzerland

**Keywords:** KRAS-driven tumors, lipid metabolism, tumor vulnerabilities, cancer therapy

## Abstract

*KRAS* is one of the most commonly mutated genes, an event that leads to development of highly aggressive and resistant to any type of available therapy tumors. Mutated *KRAS* drives a complex network of lipid metabolic rearrangements to support the adaptation of cancer cells to harsh environmental conditions and ensure their survival. Because there has been only a little success in the continuous efforts of effectively targeting KRAS-driven tumors, it is of outmost importance to delineate the exact mechanisms of how they get rewired, leading to this distinctive phenotype. Therefore, the aim of this review is to summarize the available data acquired over the last years with regard to the lipid metabolic regulation of KRAS-driven tumors and elucidate their specific characteristics in an attempt to unravel novel therapeutic targets.

## 1. Introduction

Altered metabolism is a well-established hallmark of cancer as a response of tumor cells to the continuously changing environmental conditions such as reduced oxygen availability (hypoxia) and nutrient deprivation. Lipid metabolism is a field of study that has recently been in the limelight, as its dysregulation allows cancer cells to meet their high energy and growth demands. One of the most commonly mutated gene in human cancer is the small GTPase *RAS*. KRAS-driven tumors, shift their metabolism towards a more anabolic profile by upregulating the expression of multiple rate-limiting enzymes involved in key metabolic processes essential for survival. With this metabolic shift, KRAS-driven tumors not only are able to produce key lipid mediators establishing an immunosuppressive tumor microenvironment (TME), but are also able to take full advantage of exogenous lipids produced by the TME such as fatty acids (FAs) prostaglandins and other lipid mediators, thus maintaining a vicious cycle that allows tumor growth and metastasis. Therefore, mutated KRAS, which is mostly encountered in pancreatic, lung and colorectal cancers, is considered a synonym for highly aggressive, resistant to conventional therapies tumors, thus leading to a devastatingly poor patient survival. Despite continuous effort, the GTPase has not been successfully targeted yet, with the only bright exception being the recent FDA approval of sotorasib (AMG 510), a small molecule targeting directly KRAS^G12C^. Thus, it is of outmost importance that we unravel unique characteristics of these tumors that could potentially be used as therapeutic vulnerabilities. To this end, in this review, we summarize and discuss the recent insights in the deregulation of lipid metabolism of KRAS-driven tumors, as well as the interplay between specific lipids and key cancer machineries.

## 2. Fatty Acid Uptake, Biosynthesis, and Degradation

The highly conserved process of FA synthesis incorporates carbons from acetyl-CoA into growing FAs, thus providing the cell with the required substrate for cell growth signaling, membrane building, and energy storage. In the 50′s, a seminal observation was made directly linking lipid biogenesis and cancer. Medes et al. described that tumor cells exhibit higher rates of lipogenesis and heavily rely on these de novo produced FAs in order to meet their increased metabolic needs [1].

Intriguingly, KRAS-driven cancers do not seem to follow this principle. Instead, these tumors scavenge serum FAs, with a preference for long-chain polyunsaturated FAs and lysophospholipids [2,3], while de novo lipid synthesis, although still present, seems to be dispensable. Pancreatic ductal adenocarcinomas (PDAC) and colorectal cancer (CRC) cells uptake FAs secreted from adipocytes, as a response to nutrient starvation, or from FAs secreted from cancer associated fibroblasts and inhibition of this process by blocking CD36 -a FA transporter on the cell surface reduces their metastatic capacity [4,5,6]. Interestingly, decreased *REDD1* expression, a stress response gene which is associated with peroxisome proliferator activated receptor γ (PPARγ)/CD36 activation, predicts poor outcomes selectively in *KRAS* mutant but not *KRAS* wild-type human lung and pancreatic adenocarcinomas [7]. Notably, in the context of mutant *KRAS*, *REDD1* loss is associated with suppressed FA synthesis, conferring resistance to acetyl-CoA carboxylase (ACC) inhibitors [7]. Therefore, CD36 is an attractive target for the treatment of *KRAS* mutant tumors.

The available data regarding whether de novo FA synthesis can be considered as a distinctive vulnerability for *KRAS* mutated tumors are, to say the least, divisive. The presence of multiple rate-limiting enzymes in the biosynthetic process creates many potential targets. The first step in lipid biosynthesis is catalyzed by ATP-citrate lyase (ACLY), a metabolic enzyme that cleaves cytoplasmic citrate, thus generating acetyl-CoA. Recently, Carrer et al. elegantly demonstrated in a *Pdx1-Cre/Kras^G12D^* mouse model that pancreas-specific ablation of ACLY suppressed acinar-to-ductal metaplasia, suggesting a role in early pancreatic tumorigenesis [8].

ACC is another gatekeeper in the FA synthesis process, responsible for malonyl-CoA production. In PDAC, ACC inhibitors blocked cancer cell proliferation and suppressed tumor growth by simultaneously downregulating the WNT and Hedgehog signaling pathways [9]. Moreover, inhibition of the ACC enzymes ACC1 and ACC2 in a preclinical model of Kras^G12D^-driven non-small cell lung cancer (NSCLC), led to reduced tumor growth [10]. However, as stated above, *KRAS* mutant tumors heavily rely on extracellularly derived lipids, therefore it is likely that ACC1/2 inhibition may not have robust antitumor effects. This is also confirmed by the authors finding that ACC1 knockout cell lines could survive only when supplied with exogenous palmitate [10].

Regarding fatty acid synthase (*FASN*), increased expression is often reported in many different cancer types and has in fact been linked to gemcitabine resistance in KRAS mutated PDAC [11]. The implicated signaling pathways vary in a tissue-specific manner. Ventura and colleagues proposed that pharmacological inhibition of FASN with TVB-3166 reduces cell proliferation and xenograft tumor growth of *KRAS* mutated NSCLC, but not CRC cell lines via, at least in part, inhibition of the PI3K–AKT–mTOR pathway [12]. This claim is in part supported by two other recent reports where it is shown that *KRAS* mutated NSCLCs exhibit increased FASN levels and its inhibition results in decreased tumor formation [13,14]. In PDAC, FASN inhibition led to decreased cancer cell proliferation and increased apoptosis [15]. To date, attempts of pharmacologically targeting FASN have been challenged by severe toxicity and compensatory mechanisms activated by cancer cells, namely upregulation of FA uptake [16]. One compound, TVB-2640, is currently in a phase 2 clinical trial in NSCLC patients bearing *KRAS* mutations (NCT03808558).

Once generated, FAs undergo sequential desaturation steps, a process performed mainly by stearoyl-CoA desaturase (SCD). However, accumulating evidence suggested that *KRAS* mutant cancer cells bypass sensitivity to SCD inhibition by scavenging unsaturated fatty acids from lysophospholipids [3]. Moreover, recent evidence showed that *KRAS* mutant cancer cells are also able to desaturate palmitate to sapienate via fatty acid desaturase 2 (FADS2), an event that confers additional independence to SCD-mediated FA desaturation [17]. Of note, dual inhibition of the desaturases induces cell death [17], underscoring the therapeutic potential of such a multi-targeting approach.

For FA synthesis to take place unobstructed, vital secondary metabolites are required, which become available through glycolysis. Therefore, attenuating the glycolytic machinery could prove to be beneficial by restricting not only the energy supply, but also the availability of indispensable, for lipid generation, metabolites. In this direction, the importance of glucose transporters in Kras-driven lung adenocarcinomas has been investigated, revealing that in a *Kras^LSL-G12D/WT^;Trp53^flox/flox^* lung adenocarcinoma mouse model concomitant deletion of the glucose transporters *Glut1* and *Glut3*, significantly decreases tumor progression [18]. On a similar note, pharmacologic inhibition of an alternative glucose transport system, the sodium-dependent glucose transporter Sglt2, delayed the development of lung adenocarcinomas [19]. Taking into account the fact that compounds for Sglt2 inhibition are already being used in the clinic to treat diabetes, it is realistic to say that targeting the lipid metabolism of cancer cells through the glycolytic pathway may become possible in the near future for the subset of patients with tumors that are heavily dependent on glycolysis.

Several key enzymes of the FA synthesis process undergo transcriptional regulation by sterol regulatory element-binding protein 1 (SREBP1), including *FASN* [20,21]. Mutant *KRAS* regulates SREBP1 mainly by upregulating the PI3K/Akt/mTOR signaling pathway [22] As expected, SREBP1 has been found upregulated in *KRAS* mutant PDAC patients and its expression correlates with poorer prognosis [23]. NSCLC cells also exhibit increased *SREBP1* levels; however, a novel, non-canonical role has recently been attributed to the gene, as it was shown that its ablation inhibits tumor growth, not by affecting lipogenesis, but by altering mitochondrial function and impairing oxidative phosphorylation [24]. Similarly, in CRC cells, genetic inhibition of either *SREBP1* or *SREBP2* leads to reduced tumor initiation and growth in vitro and in vivo, accompanied by decreased oxidative phosphorylation and glycolysis levels [25]. Hence, it would undoubtedly be of interest to investigate whether SREBP, apart from its well-established role in FA and cholesterol synthesis, is also directly regulating genes involved in mitochondrial function in a setting of KRAS-driven tumorigenesis.

On the other side of the spectrum, FA degradation by β-oxidation (FAO) is responsible for energy supply and NADPH production. For years, the synthesis and degradation of FAs were considered as two mutually exclusive cellular processes. The tide has turned over the last decade, and we are now at a point where it is accepted that both pathways can, in fact, be active concomitantly and independently [26,27]. FAO is tightly regulated by carnitine palmitoyltransferase 1 (CPT1), an enzyme that couples FAs with carnitine to enable their translocation to the mitochondria. Increased FAO levels directly correlate with accelerated progression and therapy resistance in KRAS-driven tumors. This phenotype can be established via multiple mechanisms. In *KRAS* mutant NSCLC, FAO is facilitated by the upregulation of long-chain acyl-CoA synthetase 3 (ACSL3), the enzyme responsible for activating free FAs, allowing tumor cells to meet the increased ATP demand [2]. Moreover, loss of *REDD1* in *KRAS* mutant NSCLC not only increases FAO, but also allows cancer cells to cope with increased reactive oxygen species (ROS) by boosting ROS detoxifying systems [7]. Interestingly, both *ACSL3* upregulation and *REDD1* loss exhibit enhanced uptake of lysophospholipids and lipid storage. Notably, in a Kras^G12D^-driven PDAC mouse model, inhibition of FAO reduces the tumor-initiating potential of a dormant, oncogene ablation-resistant, population of tumor cells that is often linked to relapse [28]. In colorectal cancer, elevated FAO is associated with extracellular FA-mediated AMPK activation, an event that strongly favors FAs as the main mitochondrial substrate for energy production [4]. Conversely, we recently reported that in hypoxic *KRAS* mutant tumors, phospholipase C γ1 (PLCγ1) is suppressed, a phenomenon leading to impaired mitochondrial fitness and decreased FAO, which boosts tumor growth [29]. Taken together, these results highlight the level of metabolic heterogeneity among hypoxic and normoxic *KRAS* mutant tumors and the imperative need to carefully characterize them on a single-cell level in our pursuit of designing effective therapeutic strategies.

**Figure 1:**
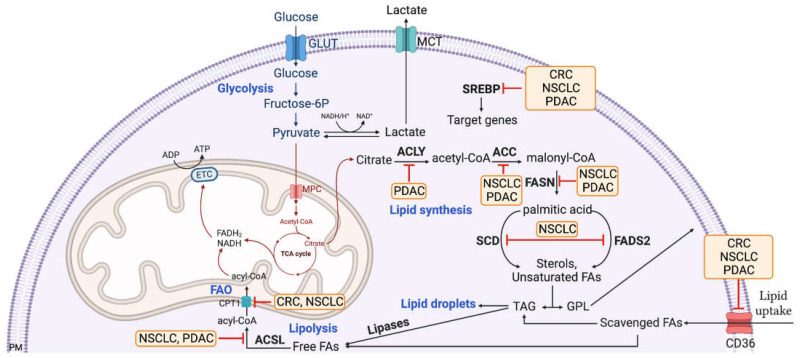
Schematic representation of FA uptake, synthesis and oxidation and the related actionable targets in different KRAS-driven cancer types. The red arrows indicate actionable targeted approaches that result in reduced tumor growth/increased cell death. The cancer types where these strategies are successful are highlighted in orange. PM: plasma membrane; CRC: colorectal cancer; NSCLC: non-small cell lung cancer; PDAC: pancreatic ductal adenocarcinomas; SCD: stearoyl-CoA desaturase; FADS2: fatty acid desaturase 2; GLUT: glucose transporter; MCT: monocarboxylate transporter; ACLY: ATP citrate lyase; ACC: acetyl-CoA carboxylase alpha; SREBP: sterol regulatory element binding transcription factor; TAG: triacylglycerols; GPL: Glycerophospholipids; CPT1: carnitine palmitoyltransferase 1A; FAO: fatty acid oxidation; ACSL: acyl-CoA synthetase long chain family members.

Taken altogether, targeting the lipid synthesis and degradation machinery in a KRAS-mutated context is a complicated concept, as there are multiple aspects to be considered (Figure 1). However, targeting the enzymes and pathways involved in FA scavenging and utilization will surely set the ground for developing new cancer treatments.

## 3. Autophagy

The role of autophagy in cancer initiation and progression, as well as therapeutic targeting has been extensively described in many excellent reviews [30,31], but the general consensus is that its complex function acts in favor of the tumors. In KRAS-driven malignancies, the autophagic machinery is commonly upregulated [32]. However, tumor initiation and progression are affected differently, with the former being inhibited and the latter promoted by autophagy. Indeed, in a Kras^G12D^-driven PDAC mouse model, genetic ablation of autophagy results in increased tumor initiation, however, these tumors are lacking the ability to progress into adenocarcinomas, thus leading to improved survival irrespective of the p53 tumor suppressor status [33]. In Kras^G12D^-driven NSCLC, loss of autophagy via Atg7 deletion before lung tumor onset did not affect tumor growth, while blocked tumor progression in mice with preexisting lung cancer [34].

In PDAC cells, an interesting crosstalk has been unveiled between pancreatic stellate cells (PSCs) and cancer cells. In a nutrient-deprived context, PDAC cells stimulate autophagy in PSCs, and this leads to secretion of alanine. Alanine is then uptaken by PDAC cells and used as alternative fuel for the TCA cycle, thus sustaining mitochondrial oxygen consumption and lipid synthesis and promoting growth during tumor initiation, but not progression [35]. The complexity of the interplay between tumor cells and the TME has also been elegantly shown in a recent work revealing that, upon induction of autophagy as a response to oxidative stress, KRAS^G12D^ protein is secreted from PDAC cells and uptaken by macrophages. This event further stimulates FAO, promotes pro-tumor macrophage M2 polarization and facilitates tumor progression [36]. The above results suggested that inhibition of autophagy could be highly effective in suppressing PDAC growth. However, this was not the case as clinical trials observed limited to no efficacy of hydroxychloroquine, an orally administered FDA-approved autophagy inhibitor [37]. 

When it comes to the control of lipid metabolism, autophagy seems to be critical for maintaining fat and glycogen stores in mice both during fasting and fed state. Indeed, systemic *Atg7* ablation leads to depletion of white adipose tissue in mice [34]. Moreover, inhibition of autophagy in CRC cells thwarts their ability to use FAs and hinders their proliferation, suggesting that mutant *KRAS* controls lipid fate by modulating the autophagic machinery [4]. Similarly, our group found that autophagy is crucial for the turnover of lipids from intracellular lipid stores (i.e., lipid droplets) in *KRAS* mutant pancreatic cancer. In a lipid-deprivation context, autophagy increases to provide cancer cells with the necessary lipids in *KRAS* mutant tumors, but not in healthy lung tissue. Indeed, concurrent extracellularly derived lipid depletion and autophagy inhibition increases cell death and suppresses PDAC progression [38]. Therefore, lipid deprivation could be exploited to render *KRAS* mutant PDAC tumors responsive to autophagy inhibitors.

## 4. The ATX-LPA Signaling Axis

Lysophosphatidic acid (LPA) is a phospholipid consisting of a glycerol backbone, a phosphate group and a FA chain that can vary in length and saturation [39]. LPA acts through LPA receptors, which are G-protein-coupled receptors, thus initiating signaling cascades involved in key cellular processes such as proliferation, survival, cytoskeletal changes, and calcium signaling [40,41]. LPA is produced mainly through two different pathways. Firstly, LPA can be generated via conversion of lysophosphatidylcholine (LPC) by autotaxin (ATX), a secreted glycoprotein that has lysophospholipase D (lysoPLD) activity [42]. Alternatively, phosphatidic acid (PA) can get deacylated by phospholipase A_1_ or A_2_ (PLA_1,_ PLA_2_) [43].

Numerous in vitro and in vivo studies suggest that alterations in the levels of LPA or its receptors are involved in tumorigenesis. By far the largest body of research, with regard to the LPA signaling axis, focuses on LPA1 and LPA3 receptors. It has been shown that in PDAC cells treated long-term with cisplatin, the activity of LPA1 and LPA receptors increased cell motility and invasion capacity, as well as MMP-2 activation [44]. These advantages could be ablated by LPA1 and LPA3 receptor knockdown, indicating that the two receptors strongly contribute to the malignant phenotype that cells acquire during tumor progression in PDAC. The same work also suggested that increased cell motility of the long-term cisplatin treated cells is, at least in part, regulated by extracellular LPA produced by ATX [44]. On a similar note, PANC-R9 cells (a highly invasive cell line established from PANC-1 pancreatic cancer cell line) were found to have higher expression levels of *LPA1* receptor compared to the parental cell line, while the expression level of *LPA3* receptor was decreased. Interestingly, the invasive capacity was amplified by LPA only in the PANC-R9 cells, an event that was accompanied with increased expression level of *ATX* [45]. Recently, it has been revealed that PDAC cells uptake FAs and particularly LPC derived from PSCs, which they then use either for membrane synthesis, or as precursors to produce signaling lipids, mainly LPA, through ATX-mediated LPC hydrolysis. Of note, ATX is overexpressed in human PDAC and its inhibition, either genetically or pharmacologically, suppressed tumor growth in an orthotopic PDAC mouse model [46]. LPA has also been involved in PDAC cell chemotaxis and metastasis in vivo [47]. Key player of this regulation is the Neural Wiskott-Aldrich Syndrome Protein (N-WASP), which drives LPA1 receptor recycling and prevents its degradation. Overall, these findings imply that the two LPA receptors, as well as LPA per se, may have a direct impact in tumor aggressiveness and resistance to drugs and their targeting could simultaneously thwart multiple hallmarks of cancer, while sparing normal cells.

The connection between the expression of LPA receptors and drug resistance has also been investigated in a CRC setting. DLD1 cells that underwent long term treatment with fluorouracil (5-FU) exhibit a robust increase in the expression levels of *LPA1* receptor. Conversely, cisplatin treatment led to a markedly elevated expression of *LPA6* receptor [48]. Using the same CRC cell line model, Shida et al. showed that LPA through LPA1 receptor stimulates cell migration, proliferation, adhesion, and the secretion of both VEGF and -in a dose dependent manner- IL-8, boosting their metastatic potential. The mechanism of LPA-induced IL-8 secretion, included degradation of IκBα and consequent activation of NF-κB [49]. 

Similarly, in a Kras^G12D^–driven lung adenocarcinoma mouse model, genetic deletion of ATX or LPA1 receptor suppressed lung tumorigenesis, confirming a pro-tumorigenic role of LPA signaling also in this context [50]. Notably, ATX was recently found to modulate also the TME because it can have a chemorepulsive action on tumor-infiltrating lymphocytes and circulating CD8+ T cells [51].

Taken altogether, it is becoming evident that KRAS-driven tumors heavily rely on the ATX-LPA signaling axis to acquire and maintain their aggressive and often therapy resistant phenotype. The mechanisms through which this is achieved seem to be cell line dependent and dictates further research. The heterogeneity in the expression pattern of LPA receptors across the different cancer types acts once more as a reminder that a “one size fits all” approach cannot be applied when planning a treatment strategy. It remains to be investigated why certain tumors rely on different LPA receptors, or their inhibition, to promote functions that will allow their propagation.

## 5. The Role of Polyunsaturated Fatty Acids (PUFAs) and Cholesterol

In view of the dependency of KRAS-driven tumors on exogenous lipids, when designing approaches to target lipid signaling it is important to consider the impact of the different extracellularly derived lipids on *KRAS* mutant cancer cells. During the last decades, a strong link between diet and cancer has been established, with high fat diet promoting tumor initiation, progression and, eventually, poorer survival [52]. Intriguingly, high calorie diet-induced obesity which leads to hypertrophic tumor-associated adipocytes in mouse and human PDAC setting, seems to increase the incidence and accelerate tumorigenesis by increasing inflammation [53,54,55]. However, in Kras^G12D^-driven lung cancer, a high calorie diet dampens tumor progression if given before tumor onset, by causing defective unfolded protein response (UPR) and unresolved endoplasmic reticulum (ER) stress, but increases tumor burden when administered after tumor onset [56]. Whether the timing of high calorie diet is important for tumor development and the exact underpinning mechanisms remain to be investigated, however, ER chaperones are unveiled as an attractive way to selectively target KRAS-driven lung tumors.

**Figure 2:**
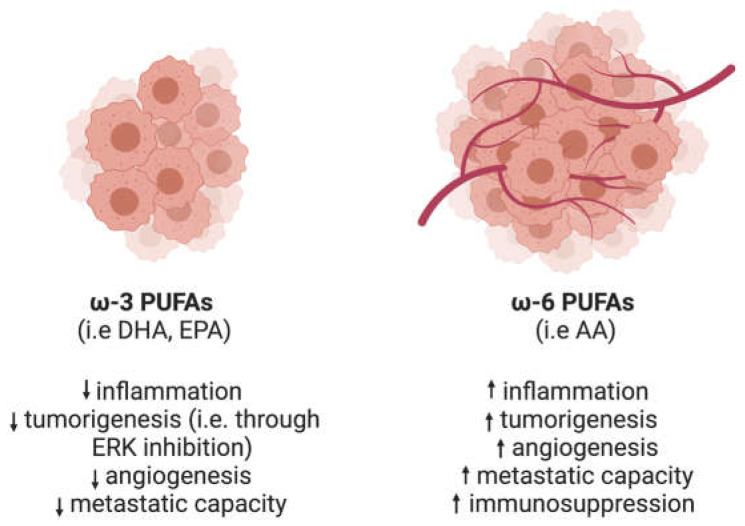
The effect of ω-3 and ω-6 PUFAs on tumor progression. ω-3 PUFAs, such as DHA or EPA, can exert an anti-tumor effect. Increased dietary uptake of these lipids has been linked to decreased inflammatory status and tumorigenesis. This is achieved through inhibition of pro-proliferative mechanisms, including the ERK pathway, inhibition of angiogenesis and decrease of the metastatic capacity. Conversely, these pathways are boosted by a diet rich in ω-6 PUFAs, such as arachidonic acid (AA).

Although the role of specific FAs in different human cancer types is not fully unraveled yet, it is becoming clear that a high uptake of ω-3 PUFAs, such as docosahexaenoic acid (DHA) and eicosapentaenoic acid (EPA), can reduce the risk of initiation and progression of some cancers including colorectal, prostate, and breast cancer (Figure 2) [57,58,59,60,61]. In fact, Collet et al. initially proposed a direct reduction of RAS localization to the plasma membrane by DHA [62]. However, it was later shown that incorporation of DHA and EPA into the plasma membrane phospholipids suppresses phosphatidic acid-dependent oncogenic KRAS-driven effector interactions (i.e., ERK signaling), thus suppressing hyperproliferation of cancer cells in vitro and in vivo [63]. Therefore, DHA rises as a promising compound that could be used to treat RAS-driven cancers.

Accumulating data obtained from both in vitro and in vivo studies point towards a direct cytotoxic effect of certain PUFAs on cancer cells [64,65,66]. This is because certain phosphoglycerolipids (mainly phosphatidylethanolamines) containing polyunsaturated acyl chains are subjected to either oxidation by ROS or selective oxygenation by lipoxygenases (LOX), a process named lipid peroxidation, which leads to ferroptosis (an iron-dependent, non-apoptotic cell death induced by the accumulation of lipid hydroperoxides) [67,68,69]. Therefore, *KRAS* mutant tumors are equipped with potent antioxidant systems to be able to overcome the cytotoxic effects of PUFAs and generally survive in the presence of high ROS levels [70]. In this context, the glutathione peroxidase 4 (GPX4) is an important ferroptosis regulator as it converts toxic lipid hydroperoxides to non-toxic lipid alcohols at the expense of glutathione (GSH) [71]. This dependency exposes a cancer cell vulnerability, in which inhibition of GPX4 either directly or indirectly with erastin (a compound which inhibits the import of cystine, leading to GSH depletion and inactivation of GPX4) results in excessive lipid peroxidation causing cancer cell death [72]. Indeed, with the view of the ongoing effort to develop drugs that can efficiently target *KRAS* mutated cancer cells, there are many recent reports that successfully leveraged the ferroptosis machinery to promote cell death in KRAS-driven tumors [73,74,75].

Furthermore, PUFAs activate PPARs, alter the expression pattern of oncogenes or oncosuppressors, and induce DNA damage [76,77,78]. With certainty, gaining better insight into the different cell functions that PUFAs can rewire will be a huge step forward in our efforts to design novel compounds and effectively tackle hard to treat malignancies.

As stated above, *KRAS* mutant cancer cells can induce the transcription of *SREBP*s, promoting cholesterol synthesis and uptake [22]. Moreover, increased *LDL-R* expression has been observed in all stages of *KRAS* mutant pancreatic cancer and is associated with increased disease recurrence rates. LDL-R silencing reduces the proliferative and clonogenic capacity of PDAC cells established from *Pdx1-Cre;LSL-Kras^G12D^;Ink4a/Arf^flox/flox^* tumors [79]. LDL-R depletion also improves the response of cells to gemcitabine and leads to tumor regression, shedding light to not only an appealing target for therapeutic exploitation, but also to a potentially effective way of sensitizing cancer cells to conventional treatment regiments [79]. In sharp contrast, treatment with statins (drugs that lower LDL blood levels) accelerates PDAC growth of *Pdx1-Cre;LSL-Kras^G12D^;Trp53^flox/flox^* mice through TGF-β induction and epithelial to mesenchymal transition (EMT) activation, but dramatically decreases tumor progression in *Pdx1-Cre;LSL-Kras^G12D^;Trp53^wt/flox^* mice, potentially due to a TGF-β-mediated, p53-dependent apoptosis initiation [80,81]. Nevertheless, one could argue that disrupting cholesterol uptake by cancer cells or thwarting *LDL-R* expression can be a promising therapy, at least for a subset of patients, that deserves further investigation.

## 6. Lipid Mediators of Inflammation

Inflammation has been linked with the initiation and progression of cancer, by enabling the tumor to acquire hallmark properties [82,83,84,85,86,87,88]. In adult mice, both chronic pancreatitis and mutated KRAS are required to induce preneoplastic lesions and PDAC [89], suggesting that inflammation is important to drive tumorigenesis in mice. Indeed, shift of the TME into a more tumor-permissive state, impediment of anti-tumor immunity, and enhancement of pro-tumorigenic signaling are commonly observed after chronic inflammation status [90]. Furthermore, *KRAS* mutant cancer cells produce inflammatory lipid mediators, establishing an inflammatory microenvironment [90]. In this well-orchestrated process, a pivotal role is played by different lipid molecules that act either as pro- or anti-inflammatory autacoids. The former comprise, among others, prostaglandins and leukotrienes [91]. On the other hand, specialized pro-resolving lipid mediators (SPMs) are produced locally by either epithelial, or immune cells and act as local hormones, binding to their specific receptors and thus initiating resolution of inflammation and tissue restoration [92]. From that perspective, SPMs have been shown to be able to modulate angiogenesis, stimulate efferocytosis, and mitigate the inflammatory response [91].

Unresolved inflammation and consequently increased risk of cancer initiation can be due to either sustained production of pro-inflammatory mediators, or lack of pro-resolving activity. It is therefore reasonable that targeting the inflammatory machinery attracted a lot of scientific attention as a promising therapeutic approach to combat cancer.

### 6.1. The Pro-Inflammatory Front: Prostaglandins

Prostaglandins, which derive from ω-6 PUFAs, namely arachidonic acid, act in a tumor promoting way both by directly activating signaling pathways, which control cancer cell proliferation, anchorage-independent growth, migration, apoptosis and angiogenesis, and by establishing an immunosuppressive TME (Figure 2) [93,94]. Despite extensive research, the exact molecular mechanisms through which tumor growth is promoted remain elusive. However, this is slowly changing, particularly with regard to prostaglandin E_2_ (PGE_2_) and how its biosynthesis pathway is involved in many different aspects of cancer progression (Table 1).

In a CRC orthotopic mouse model, PGE_2_ increased the invasiveness and metastatic capacity of cancer cells by elevating the expression levels of *miR675-5p*, which in turn binds to *p53* mRNA and prevents its translation [95]. In contrast, in a clinical study conducted by Zhang et al., a robust decrease in serum concentration of PGE_2_ and its product, 20-hydroxy-PGE_2,_ were observed in CRC patients compared to the healthy cohort [96].

Accumulation of PGE_2_ can be due to either increased biosynthesis, or decreased degradation through 15-hydroxyprostaglandin dehydrogenase (15-PGDH), the enzyme responsible for degrading prostaglandins. In *Kras^G12D^* -driven lung adenocarcinomas, the production of prostaglandins PGE_2_, PGD_2_, and PGI_2_ is greatly increased compared to healthy lungs. This increase is ACSL3 dependent as *Kras^G12D^*;*Acsl3^−/−^* lung tumors evidence impaired prostaglandin production. Increased prostaglandin synthesis requires sufficient amounts of their precursor arachidonic acid. How *KRAS* mutant cancer cells manage to sustain such high levels of arachidonic acid to be able to sustain the prostaglandin production is not well understood. Notably, the lysophosphatidylinositol-acyltransferase 1 (LPIAT1), which is involved in reacylation of phospholipids with specificity for arachidonic acid, is overexpressed in *KRAS* mutant lung cancer [97]. We recently shed light to this mechanism by determining that ACSL3 channels arachidonic acid to LPIAT1 to boost the pool of arachidonic acid-bound phosphatidylinositol (PI) and consequently accommodate the high prostaglandin production rates [97]. Alternatively, the strongly upregulated *miR-574-5p* directly antagonizes *CUGBP1*, a negative regulator of PGE synthase-1 (PGES-1), allowing the expression of an alternative *PGES-1* 3′UTR variant, increasing PGE_2_ production and promoting lung tumor growth in vivo [98].

In PDAC, *15-PGDH* expression is inversely correlated with *ALDH1*, a well described cancer stem cell (CSC) marker and pharmacological inhibition of 15-PGDH leads to the expansion of the ALDH1-positive PDAC cells in vitro and in vivo [99]. Moreover, interleukin-1β-mediated decrease of 15-PGDH is often observed in PDAC and is associated with poor prognosis [100].

The arachidonic acid/PLA_2_/COX2/PGE_2_ synthesis pathway can also get stimulated in cancer cells by the TME. In this context, IL-6 is secreted by macrophages in the TME, an event that induces the translocation of β-catenin from the cytoplasm to the nucleus, leading to EMT and increased invasiveness of lung cancer cells [101]. Once produced, PGE_2_ upregulates the levels of PD-1 in infiltrating CD8^+^ T cells via EP2 and EP4, resulting in immune tolerance in the lung cancer TME [102]. Lastly, further highlighting the complexity of the prostaglandins production pathway, a recent study using an in vitro *KRAS* mutant NSCLC model showed that, unlike COX2, inhibition of PGES creates a pro-cell death state which coincides with accumulation of sphinganine and dihydroceramide C16:0, two sphingolipid species associated with cancer cell death [103]. Therefore, these data suggest that *KRAS* mutant cancer cells crosstalk with the TME to sustain their high prostaglandin demand, leading to tumor progression and invasion.

Taken together, the above data underscore the therapeutic potential of suppressing prostaglandin production not only in cancer cells but also in components of the TME.

### 6.2. Pro-Resolving Lipid Mediators

The most thoroughly investigated SPM is lipoxin A4 (LXA_4_), an anti-inflammatory metabolite derived from AA. In pancreatic cancer, LXA_4_ is associated with attenuated cancer cell invasion and metastasis by inhibiting KRAS-induced ROS production, as well as ERK/MMPs and TGF-β1 signaling pathways [104,105]. Furthermore, LXA_4_ can modulate different elements of the TME in various cancer models. In CRC, LXA_4_ has a tumor suppressing role by targeting, at least in part, regulatory B-cells and as a result facilitating the activity of CD8^+^ cytotoxic T cells in the TME [106].

In addition to LXA_4_, resolvins also have therapeutic potential. In a pancreatic cancer model, RvD1 did not only modify the TME by supporting the cytotoxic activity of NK cells, but it also induced tumor cell apoptosis [107]. In a lung cancer setting, RvD1 and RvD2 suppressed the TGF-β1-induced EMT in vitro, further supporting the fact that these lipid mediators exert key anti-tumor functions that can also be independent of their anti-inflammatory properties [108].

A few years back, aspirin was in the spotlight about its potential anti-tumor effects. What sets aspirin apart from other NSAIDs is the fact that, apart from blocking prostaglandins synthesis by inhibiting the pro-inflammatory COX pathway, is also responsible for stimulating the production of anti-inflammatory mediators, such as aspirin-triggered lipoxins (AT-LXs) and resolvins (AT-RvDs), albeit rather weakly [109,110]. In fact, treatment with AT-LXA_4_, or AT-RvDs strongly reduces tumor growth in a Lewis lung carcinoma cell (LLC; *KRAS^G12C^* mutated) -derived lung cancer mouse model, by negatively regulating the secretion of pro-inflammatory cytokines including macrophage migration inhibitory factor (MIF), plasminogen activator inhibitor-1 (PAI-1), and C-C motif chemokine ligand 2 (CCL2) and stimulating macrophage phagocytosis of tumor cell debris [111]. The substantially lower concentration required to achieve these effects means that by directly targeting the resolvin pathways, the aspirin-induced toxicity and immunosuppression no longer poses a limitation (Table 1).

## 7. Conclusions

Over the last years, a growing body of evidence substantiated the key role of lipid metabolism in carcinogenesis. Thus, it is now widely accepted that cancer cells display differential regulation of lipid metabolism, thus affecting tumor progression, invasion, and metastasis through an extremely dynamic and complex signaling network. Against this backdrop, developing novel drugs that will successfully target lipid metabolism, to prevent or efficiently treat cancer, is an attractive prospect. Therefore, understanding how cancer cells adapt their metabolism to meet their high energy and biosynthetic needs is of outmost importance. For instance, even though FA synthesis and FAO were once considered as mutually exclusive, it is nowadays known that both cellular processes can, in fact, be concomitantly active.

KRAS-driven cancers are notorious for their aggressiveness and resistance to therapy. It is therefore tempting to speculate that they bear a specific lipid metabolic “fingerprint”, the identification of which will allow us to efficiently target them. It is becoming apparent that apart from synthesizing lipids de novo, those tumor cells are also able to uptake lipids, sometimes even by stimulating their secretion from the surrounding tissue, underscoring once more the importance of detailed investigation of the TME. The excellent work of various research groups that was summarized in this review, indicates that tumors bearing oncogenic KRAS rewire their lipid metabolism in such a tissue- and context-specific way that allows them to adapt to any conditions. This is manifested in many different cellular functions, including FA synthesis, FAO, autophagy, and inflammation. It is evident that in our pursuit of a better understanding of these tumors, we need to evaluate the available preclinical and clinical data not only in a tissue-, but also oncosuppressor- and time-specific manner.

## Figures and Tables

**Table 1:** Effect of prostaglandins in different cancer types and the implicated driving mechanisms.

Cancer Type	Model Used	Reported Effect	Involved Mechanism
CRC	Orthotopic mouse model	Pro-tumorigenic; ↑ invasiveness; ↑ metastatic capacity	↑ miR675-5p, ↓ p53 translation [95]
NSCLC	In vitro *Kras*^LSL*-G12D*/+^*;Trp53^fl/fl^* mice	Pro-tumorigenic; ↑ tumor cell proliferation; ↑ anchorage independent growth	ACSL3-mediated AA channeling to PI, as substrate for prostaglandin production [97]
NSCLC	In vitro; clinical samples; xenograft model	Pro-tumorigenic; ↑ tumor growth	↑ miR-574-5p, ↓ CUGBP1, ↑ mPGES-1 [98]
NSCLC	In vitro; clinical samples	Pro-tumorigenic; ↑ EMT; ↑ invasiveness	IL-6-mediated COX-2/PGE_2_/β-catenin pathway activation [101]
NSCLC	Clinical samples	Pro-tumorigenic; ↑ immunotolerance	EP2- and EP4-mediated increase of PD-1 in infiltrating CD8^+^ T cells [102]
NSCLC	In vitro;	↓ cell proliferation ↑ cytotoxic effect of cisplatin, etoposide, and vincristine	mPGES1 inhibition [103]
PDAC	In vitro; *Kras*^LSL*-G12D*/+^*; Ptf1a^Cre/+^* mice	Pro-tumorigenic; ↑ tumor cell growth; ↑ sphere formation through activation of ALDH1	EP2- and EP4-mediated ALDH1 activation [99]
PDAC	In vitro; clinical samples	Pro-tumorigenic; ↑ tumor cell growth; ↑ lymph node metastasis; ↑ nerve invasion; poor prognosis	IL-1β-mediated ↓ 15-PGDH [100]

## Data Availability

Not applicable.

## Abbreviations

CRC: colorectal cancer; NSCLC, non-small cell lung cancer; PDAC, pancreatic ductal adenocarcinomas; ACLY, ATP-citrate lyase; ACC, FASN, fatty acid synthase; Acetyl-CoA carboxylase; FA, fatty acid; SCD, stearoyl-CoA desaturase; FADS2, fatty acid desaturase 2; SREBP1, sterol regulatory element-binding protein 1; CPT1, carnitine palmitoyltransferase 1; ROS, reactive oxygen species; FAO, fatty acid oxidation; PSCs, pancreatic stellate cells; TME, tumor microenvironment; PUFAs, polyunsaturated fatty acids; UPR, unfolded protein response; ER, endoplasmic reticulum; DHA, docosahexaenoic acid; EPA, eicosapentaenoic acid; LA, linoleic acid; AA, arachidonic acid; LDL-R, low-density lipoprotein receptor; LOX, lipoxygenases; GPX4, glutathione peroxidase 4; PPARs, peroxisome proliferator activated receptors; LPA, lysophosphatidic acid; ATX, autotaxin; N-WASP, neural wiskott-aldrich syndrome protein; ALDH1: aldehyde dehydrogenase 1 family, member A1; LXA_4_, lipoxin A4; SPM, pro-resolving lipid mediators; PGE_2_, prostaglandin E_2_.

## References

[1] Medes G., Thomas A., Weinhouse S. (1953). Metabolism of neoplastic tissue. IV. A study of lipid synthesis in neoplastic tissue slices in vitro. Cancer Res..

[2] Padanad M.S., Konstantinidou G., Venkateswaran N., Melegari M., Rindhe S., Mitsche M., Yang C., Batten K., Huffman K.E., Liu J. (2016). Fatty Acid Oxidation Mediated by Acyl-CoA Synthetase Long Chain 3 Is Required for Mutant KRAS Lung Tumorigenesis. Cell Rep..

[3] Kamphorst J.J., Cross J.R., Fan J., de Stanchina E., Mathew R., White E.P., Thompson C.B., Rabinowitz J.D. (2013). Hypoxic and Ras-transformed cells support growth by scavenging unsaturated fatty acids from lysophospholipids. Proc. Natl. Acad. Sci. USA.

[4] Wen Y.-A., Xing X., Harris J.W., Zaytseva Y.Y., Mitov M.I., Napier D.L., Weiss H.L., Mark Evers B., Gao T. (2017). Adipocytes activate mitochondrial fatty acid oxidation and autophagy to promote tumor growth in colon cancer. Cell Death Dis..

[5] Gong J., Lin Y., Zhang H., Liu C., Cheng Z., Yang X., Zhang J., Xiao Y., Sang N., Qian X. (2020). Reprogramming of lipid metabolism in cancer-associated fibroblasts potentiates migration of colorectal cancer cells. Cell Death Dis..

[6] Okumura T., Ohuchida K., Sada M., Abe T., Endo S., Koikawa K., Iwamoto C., Miura D., Mizuuchi Y., Moriyama T. (2017). Extra-pancreatic invasion induces lipolytic and fibrotic changes in the adipose microenvironment, with released fatty acids enhancing the invasiveness of pancreatic cancer cells. Oncotarget.

[7] Qiao S., Koh S.-B., Vivekanandan V., Salunke D., Patra K.C., Zaganjor E., Ross K., Mizukami Y., Jeanfavre S., Chen A. (2020). REDD1 loss reprograms lipid metabolism to drive progression of RAS mutant tumors. Genes Dev..

[8] Carrer A., Trefely S., Zhao S., Campbell S.L., Norgard R.J., Schultz K.C., Sidoli S., Parris J.L.D., Affronti H.C., Sivanand S. (2019). Acetyl-CoA Metabolism Supports Multistep Pancreatic Tumorigenesis. Cancer Discov..

[9] Petrova E., Scholz A., Paul J., Sturz A., Haike K., Siegel F., Mumberg D., Liu N. (2017). Acetyl-CoA carboxylase inhibitors attenuate WNT and Hedgehog signaling and suppress pancreatic tumor growth. Oncotarget.

[10] Svensson R.U., Parker S.J., Eichner L.J., Kolar M.J., Wallace M., Brun S.N., Lombardo P.S., Van Nostrand J.L., Hutchins A., Vera L. (2016). Inhibition of acetyl-CoA carboxylase suppresses fatty acid synthesis and tumor growth of non-small-cell lung cancer in preclinical models. Nat. Med..

[11] Tadros S., Shukla S.K., King R.J., Gunda V., Vernucci E., Abrego J., Chaika N.V., Yu F., Lazenby A.J., Berim L. (2017). De Novo Lipid Synthesis Facilitates Gemcitabine Resistance through Endoplasmic Reticulum Stress in Pancreatic Cancer. Cancer Res..

[12] Ventura R., Mordec K., Waszczuk J., Wang Z., Lai J., Fridlib M., Buckley D., Kemble G., Heuer T.S. (2015). Inhibition of de novo Palmitate Synthesis by Fatty Acid Synthase Induces Apoptosis in Tumor Cells by Remodeling Cell Membranes, Inhibiting Signaling Pathways, and Reprogramming Gene Expression. EBioMedicine.

[13] Gouw A.M., Eberlin L.S., Margulis K., Sullivan D.K., Toal G.G., Tong L., Zare R.N., Felsher D.W. (2017). Oncogene KRAS activates fatty acid synthase, resulting in specific ERK and lipid signatures associated with lung adenocarcinoma. Proc. Natl. Acad. Sci. USA.

[14] Singh A., Ruiz C., Bhalla K., Haley J.A., Li Q.K., Acquaah-Mensah G., Montal E., Sudini K.R., Skoulidis F., Wistuba I.I. (2018). De novo lipogenesis represents a therapeutic target in mutant Kras non-small cell lung cancer. FASEB J..

[15] Bian Y., Yu Y., Wang S., Li L. (2015). Up-regulation of fatty acid synthase induced by EGFR/ERK activation promotes tumor growth in pancreatic cancer. Biochem. Biophys. Res. Commun..

[16] Röhrig F., Schulze A. (2016). The multifaceted roles of fatty acid synthesis in cancer. Nat. Rev. Cancer.

[17] Vriens K., Christen S., Parik S., Broekaert D., Yoshinaga K., Talebi A., Dehairs J., Escalona-Noguero C., Schmieder R., Cornfield T. (2019). Evidence for an alternative fatty acid desaturation pathway increasing cancer plasticity. Nature.

[18] Contat C., Ancey P.-B., Zangger N., Sabatino S., Pascual J., Escrig S., Jensen L., Goepfert C., Lanz B., Lepore M. (2020). Combined deletion of Glut1 and Glut3 impairs lung adenocarcinoma growth. eLife.

[19] Scafoglio C.R., Villegas B., Abdelhady G., Bailey S.T., Liu J., Shirali A.S., Wallace W.D., Magyar C.E., Grogan T.R., Elashoff D. (2018). Sodium-glucose transporter 2 is a diagnostic and therapeutic target for early-stage lung adenocarcinoma. Sci. Transl. Med..

[20] Shimano H., Yahagi N., Amemiya-Kudo M., Hasty A.H., Osuga J., Tamura Y., Shionoiri F., Iizuka Y., Ohashi K., Harada K. (1999). Sterol regulatory element-binding protein-1 as a key transcription factor for nutritional induction of lipogenic enzyme genes. J. Biol. Chem..

[21] Towle H.C. (1995). Metabolic regulation of gene transcription in mammals. J. Biol. Chem..

[22] Ricoult S.J., Yecies J.L., Ben-Sahra I., Manning B.D. (2016). Oncogenic PI3K and K-Ras stimulate de novo lipid synthesis through mTORC1 and SREBP. Oncogene.

[23] Sun Y., He W., Luo M., Zhou Y., Chang G., Ren W., Wu K., Li X., Shen J., Zhao X. (2015). SREBP1 regulates tumorigenesis and prognosis of pancreatic cancer through targeting lipid metabolism. Tumour Biol..

[24] Ruiz C.F., Montal E.D., Haley J.A., Bott A.J., Haley J.D. (2020). SREBP1 regulates mitochondrial metabolism in oncogenic KRAS expressing NSCLC. FASEB J..

[25] Wen Y.-A., Xiong X., Zaytseva Y.Y., Napier D.L., Vallee E., Li A.T., Wang C., Weiss H.L., Evers B.M., Gao T. (2018). Downregulation of SREBP inhibits tumor growth and initiation by altering cellular metabolism in colon cancer. Cell Death Dis..

[26] Caro P., Kishan A.U., Norberg E., Stanley I.A., Chapuy B., Ficarro S.B., Polak K., Tondera D., Gounarides J., Yin H. (2012). Metabolic signatures uncover distinct targets in molecular subsets of diffuse large B cell lymphoma. Cancer Cell.

[27] Jeon S.-M., Chandel N.S., Hay N. (2012). AMPK regulates NADPH homeostasis to promote tumour cell survival during energy stress. Nature.

[28] Viale A., Pettazzoni P., Lyssiotis C.A., Ying H., Sanchez N., Marchesini M., Carugo A., Green T., Seth S., Giuliani V. (2014). Oncogene ablation-resistant pancreatic cancer cells depend on mitochondrial function. Nature.

[29] Saliakoura M., Rossi Sebastiano M., Pozzato C., Heidel F.H., Schnoder T.M., Savic Prince S., Bubendorf L., Pinton P., Schmid A.R., Baumgartner J. (2020). PLCgamma1 suppression promotes the adaptation of KRAS-mutant lung adenocarcinomas to hypoxia. Nat. Cell Biol..

[30] Mathew R., Karantza-Wadsworth V., White E. (2007). Role of autophagy in cancer. Nat. Rev. Cancer.

[31] Levy J.M.M., Towers C.G., Thorburn A. (2017). Targeting autophagy in cancer. Nat. Rev. Cancer.

[32] Guo J.Y., Chen H.Y., Mathew R., Fan J., Strohecker A.M., Karsli-Uzunbas G., Kamphorst J.J., Chen G., Lemons J.M., Karantza V. (2011). Activated Ras requires autophagy to maintain oxidative metabolism and tumorigenesis. Genes Dev..

[33] Yang A., Rajeshkumar N.V., Wang X., Yabuuchi S., Alexander B.M., Chu G.C., Von Hoff D.D., Maitra A., Kimmelman A.C. (2014). Autophagy Is Critical for Pancreatic Tumor Growth and Progression in Tumors with p53 Alterations. Cancer Discov..

[34] Karsli-Uzunbas G., Guo J.Y., Price S., Teng X., Laddha S.V., Khor S., Kalaany N.Y., Jacks T., Chan C.S., Rabinowitz J.D. (2014). Autophagy is required for glucose homeostasis and lung tumor maintenance. Cancer Discov..

[35] Sousa C.M., Biancur D.E., Wang X., Halbrook C.J., Sherman M.H., Zhang L., Kremer D., Hwang R.F., Witkiewicz A.K., Ying H. (2016). Pancreatic stellate cells support tumour metabolism through autophagic alanine secretion. Nature.

[36] Dai E., Han L., Liu J., Xie Y., Kroemer G., Klionsky D.J., Zeh H.J., Kang R., Wang J., Tang D. (2020). Autophagy-dependent ferroptosis drives tumor-associated macrophage polarization via release and uptake of oncogenic KRAS protein. Autophagy.

[37] Wolpin B.M., Rubinson D.A., Wang X., Chan J.A., Cleary J.M., Enzinger P.C., Fuchs C.S., McCleary N.J., Meyerhardt J.A., Ng K. (2014). Phase II and pharmacodynamic study of autophagy inhibition using hydroxychloroquine in patients with metastatic pancreatic adenocarcinoma. Oncologist.

[38] Saliakoura M., Sebastiano M.R., Nikdima I., Pozzato C., Konstantinidou G. (2022). Restriction of extracellular lipids renders pancreatic cancer dependent on autophagy. J. Exp. Clin. Cancer Res..

[39] Tokumura A. (1995). A family of phospholipid autacoids: Occurrence, metabolism and bioactions. Prog. Lipid Res..

[40] Fukushima N., Ishii I., Contos J.J., Weiner J.A., Chun J. (2001). Lysophospholipid receptors. Annu. Rev. Pharmacol. Toxicol..

[41] Ishii I., Fukushima N., Ye X., Chun J. (2004). Lysophospholipid receptors: Signaling and biology. Annu Rev. Biochem.

[42] Umezu-Goto M., Kishi Y., Taira A., Hama K., Dohmae N., Takio K., Yamori T., Mills G.B., Inoue K., Aoki J. (2002). Autotaxin has lysophospholipase D activity leading to tumor cell growth and motility by lysophosphatidic acid production. J. Cell Biol..

[43] Fourcade O., Simon M.F., Viode C., Rugani N., Leballe F., Ragab A., Fournie B., Sarda L., Chap H. (1995). Secretory phospholipase A2 generates the novel lipid mediator lysophosphatidic acid in membrane microvesicles shed from activated cells. Cell.

[44] Fukushima K., Takahashi K., Yamasaki E., Onishi Y., Fukushima N., Honoki K., Tsujiuchi T. (2017). Lysophosphatidic acid signaling via LPA1 and LPA3 regulates cellular functions during tumor progression in pancreatic cancer cells. Exp. Cell Res..

[45] Fukushima K., Otagaki S., Takahashi K., Minami K., Ishimoto K., Fukushima N., Honoki K., Tsujiuchi T. (2018). Promotion of cell-invasive activity through the induction of LPA receptor-1 in pancreatic cancer cells. J. Recept. Signal. Transduct..

[46] Auciello F.R., Bulusu V., Oon C., Tait-Mulder J., Berry M., Bhattacharyya S., Tumanov S., Allen-Petersen B.L., Link J., Kendsersky N.D. (2019). A Stromal Lysolipid-Autotaxin Signaling Axis Promotes Pancreatic Tumor Progression. Cancer Discov..

[47] Juin A., Spence H.J., Martin K.J., McGhee E., Neilson M., Cutiongco M.F.A., Gadegaard N., Mackay G., Fort L., Lilla S. (2019). N-WASP Control of LPAR1 Trafficking Establishes Response to Self-Generated LPA Gradients to Promote Pancreatic Cancer Cell Metastasis. Dev. Cell.

[48] Takahashi K., Fukushima K., Otagaki S., Ishimoto K., Minami K., Fukushima N., Honoki K., Tsujiuchi T. (2018). Effects of LPA1 and LPA6 on the regulation of colony formation activity in colon cancer cells treated with anticancer drugs. J. Recept. Signal Transduct..

[49] Shida D., Kitayama J., Yamaguchi H., Okaji Y., Tsuno N.H., Watanabe T., Takuwa Y., Nagawa H. (2003). Lysophosphatidic Acid (LPA) Enhances the Metastatic Potential of Human Colon Carcinoma DLD1 Cells through LPA1. Cancer Res..

[50] Magkrioti C., Oikonomou N., Kaffe E., Mouratis M.A., Xylourgidis N., Barbayianni I., Megadoukas P., Harokopos V., Valavanis C., Chun J. (2018). The Autotaxin-Lysophosphatidic Acid Axis Promotes Lung Carcinogenesis. Cancer Res..

[51] Matas-Rico E., Frijlink E., van der Haar Avila I., Menegakis A., van Zon M., Morris A.J., Koster J., Salgado-Polo F., de Kivit S., Lanca T. (2021). Autotaxin impedes anti-tumor immunity by suppressing chemotaxis and tumor infiltration of CD8(+) T cells. Cell Rep..

[52] Willett W.C. (2001). Diet and cancer: One view at the start of the millennium. Cancer Epidemiol. Biomark. Prev..

[53] Incio J., Liu H., Suboj P., Chin S.M., Chen I.X., Pinter M., Ng M.R., Nia H.T., Grahovac J., Kao S. (2016). Obesity-Induced Inflammation and Desmoplasia Promote Pancreatic Cancer Progression and Resistance to Chemotherapy. Cancer Discov..

[54] Berrington de Gonzalez A., Sweetland S., Spencer E. (2003). A meta-analysis of obesity and the risk of pancreatic cancer. Br. J. Cancer.

[55] Calle E.E., Rodriguez C., Walker-Thurmond K., Thun M.J. (2003). Overweight, obesity, and mortality from cancer in a prospectively studied cohort of U.S. adults. N. Engl. J. Med..

[56] Ramadori G., Konstantinidou G., Venkateswaran N., Biscotti T., Morlock L., Galie M., Williams N.S., Luchetti M., Santinelli A., Scaglioni P.P. (2015). Diet-Induced Unresolved ER Stress Hinders KRAS-Driven Lung Tumorigenesis. Cell Metab..

[57] Lipworth L. (1995). Epidemiology of breast cancer. Eur. J. Cancer Prev..

[58] Potter J.D. (1995). Risk factors for colon neoplasia—Epidemiology and biology. Eur. J. Cancer.

[59] Key T. (1995). Risk factors for prostate cancer. Cancer Surv..

[60] Moro K., Nagahashi M., Ramanathan R., Takabe K., Wakai T. (2016). Resolvins and omega three polyunsaturated fatty acids: Clinical implications in inflammatory diseases and cancer. World J. Clin. Cases.

[61] Cockbain A.J., Toogood G.J., Hull M.A. (2012). Omega-3 polyunsaturated fatty acids for the treatment and prevention of colorectal cancer. Gut.

[62] Collett E.D., Davidson L.A., Fan Y.-Y., Lupton J.R., Chapkin R.S. (2001). n-6 and n-3 polyunsaturated fatty acids differentially modulate oncogenic Ras activation in colonocytes. Am. J. Physiol. Cell Physiol..

[63] Fuentes N.R., Mlih M., Barhoumi R., Fan Y.Y., Hardin P., Steele T.J., Behmer S., Prior I.A., Karpac J., Chapkin R.S. (2018). Long-Chain n-3 Fatty Acids Attenuate Oncogenic KRas-Driven Proliferation by Altering Plasma Membrane Nanoscale Proteolipid Composition. Cancer Res..

[64] Klurfeld D.M., Bull A.W. (1997). Fatty acids and colon cancer in experimental models. Am. J. Clin. Nutr..

[65] Chapkin R.S., Seo J., McMurray D.N., Lupton J.R. (2008). Mechanisms by which docosahexaenoic acid and related fatty acids reduce colon cancer risk and inflammatory disorders of the intestine. Chem. Phys. Lipids.

[66] Trombetta A., Maggiora M., Martinasso G., Cotogni P., Canuto R.A., Muzio G. (2007). Arachidonic and docosahexaenoic acids reduce the growth of A549 human lung-tumor cells increasing lipid peroxidation and PPARs. Chem. Biol. Interact..

[67] Stockwell B.R., Friedmann Angeli J.P., Bayir H., Bush A.I., Conrad M., Dixon S.J., Fulda S., Gascon S., Hatzios S.K., Kagan V.E. (2017). Ferroptosis: A Regulated Cell Death Nexus Linking Metabolism, Redox Biology, and Disease. Cell.

[68] Yang W.S., Kim K.J., Gaschler M.M., Patel M., Shchepinov M.S., Stockwell B.R. (2016). Peroxidation of polyunsaturated fatty acids by lipoxygenases drives ferroptosis. Proc. Natl. Acad. Sci. USA.

[69] Dixon S.J., Lemberg K.M., Lamprecht M.R., Skouta R., Zaitsev E.M., Gleason C.E., Patel D.N., Bauer A.J., Cantley A.M., Yang W.S. (2012). Ferroptosis: An iron-dependent form of nonapoptotic cell death. Cell.

[70] DeNicola G.M., Karreth F.A., Humpton T.J., Gopinathan A., Wei C., Frese K., Mangal D., Yu K.H., Yeo C.J., Calhoun E.S. (2011). Oncogene-induced Nrf2 transcription promotes ROS detoxification and tumorigenesis. Nature.

[71] Ursini F., Maiorino M., Valente M., Ferri L., Gregolin C. (1982). Purification from pig liver of a protein which protects liposomes and biomembranes from peroxidative degradation and exhibits glutathione peroxidase activity on phosphatidylcholine hydroperoxides. Biochim. Biophys. Acta.

[72] Yang W.S., Stockwell B.R. (2016). Ferroptosis: Death by Lipid Peroxidation. Trends Cell Biol..

[73] Yang J., Mo J., Dai J., Ye C., Cen W., Zheng X., Jiang L., Ye L. (2021). Cetuximab promotes RSL3-induced ferroptosis by suppressing the Nrf2/HO-1 signalling pathway in KRAS mutant colorectal cancer. Cell Death Dis..

[74] Bartolacci C., Andreani C., Vale G., Berto S., Melegari M., Crouch A.C., Baluya D.L., Kemble G., Hodges K., Starrett J. (2022). Targeting de novo lipogenesis and the Lands cycle induces ferroptosis in KRAS-mutant lung cancer. Nat. Commun..

[75] Hu K., Li K., Lv J., Feng J., Chen J., Wu H., Cheng F., Jiang W., Wang J., Pei H. (2020). Suppression of the SLC7A11/glutathione axis causes synthetic lethality in KRAS-mutant lung adenocarcinoma. J. Clin. Investig..

[76] Monjazeb A.M., High K.P., Connoy A., Hart L.S., Koumenis C., Chilton F.H. (2006). Arachidonic acid-induced gene expression in colon cancer cells. Carcinogenesis.

[77] Narayanan B.A., Narayanan N.K., Reddy B.S. (2001). Docosahexaenoic acid regulated genes and transcription factors inducing apoptosis in human colon cancer cells. Int. J. Oncol..

[78] Wang S., Dougherty E.J., Danner R.L. (2016). PPARγ signaling and emerging opportunities for improved therapeutics. Pharmacol. Res..

[79] Guillaumond F., Bidaut G., Ouaissi M., Servais S., Gouirand V., Olivares O., Lac S., Borge L., Roques J., Gayet O. (2015). Cholesterol uptake disruption, in association with chemotherapy, is a promising combined metabolic therapy for pancreatic adenocarcinoma. Proc. Natl. Acad. Sci. USA.

[80] Gabitova-Cornell L., Surumbayeva A., Peri S., Franco-Barraza J., Restifo D., Weitz N., Ogier C., Goldman A.R., Hartman T.R., Francescone R. (2020). Cholesterol Pathway Inhibition Induces TGF-β Signaling to Promote Basal Differentiation in Pancreatic Cancer. Cancer Cell.

[81] Cordenonsi M., Dupont S., Maretto S., Insinga A., Imbriano C., Piccolo S. (2003). Links between tumor suppressors: p53 is required for TGF-β gene responses by cooperating with Smads. Cell.

[82] Hanahan D., Weinberg R.A. (2011). Hallmarks of cancer: The next generation. Cell.

[83] Medzhitov R. (2008). Origin and physiological roles of inflammation. Nature.

[84] Dohadwala M., Batra R.K., Luo J., Lin Y., Krysan K., Pold M., Sharma S., Dubinett S.M. (2002). Autocrine/paracrine prostaglandin E2 production by non-small cell lung cancer cells regulates matrix metalloproteinase-2 and CD44 in cyclooxygenase-2-dependent invasion. J. Biol. Chem..

[85] Pai R., Soreghan B., Szabo I.L., Pavelka M., Baatar D., Tarnawski A.S. (2002). Prostaglandin E2 transactivates EGF receptor: A novel mechanism for promoting colon cancer growth and gastrointestinal hypertrophy. Nat. Med..

[86] Bernard M.P., Bancos S., Sime P.J., Phipps R.P. (2008). Targeting cyclooxygenase-2 in hematological malignancies: Rationale and promise. Curr. Pharm. Des..

[87] Young L.E., Dixon D.A. (2010). Posttranscriptional Regulation of Cyclooxygenase 2 Expression in Colorectal Cancer. Curr. Color. Cancer Rep..

[88] Denkert C., Winzer K.J., Muller B.M., Weichert W., Pest S., Kobel M., Kristiansen G., Reles A., Siegert A., Guski H. (2003). Elevated expression of cyclooxygenase-2 is a negative prognostic factor for disease free survival and overall survival in patients with breast carcinoma. Cancer.

[89] Guerra C., Schuhmacher A.J., Canamero M., Grippo P.J., Verdaguer L., Perez-Gallego L., Dubus P., Sandgren E.P., Barbacid M. (2007). Chronic pancreatitis is essential for induction of pancreatic ductal adenocarcinoma by K-Ras oncogenes in adult mice. Cancer Cell.

[90] Mantovani A., Allavena P., Sica A., Balkwill F. (2008). Cancer-related inflammation. Nature.

[91] Serhan C.N. (2014). Pro-resolving lipid mediators are leads for resolution physiology. Nature.

[92] Serhan C.N., Chiang N., Dalli J., Levy B.D. (2015). Lipid Mediators in the Resolution of Inflammation. Cold Spring Harb. Perspect. Biol..

[93] Huang M., Stolina M., Sharma S., Mao J.T., Zhu L., Miller P.W., Wollman J., Herschman H., Dubinett S.M. (1998). Non-small cell lung cancer cyclooxygenase-2-dependent regulation of cytokine balance in lymphocytes and macrophages: Up-regulation of interleukin 10 and down-regulation of interleukin 12 production. Cancer Res..

[94] Wang D., Dubois R.N. (2006). Prostaglandins and cancer. Gut.

[95] Cen B., Lang J.D., Du Y., Wei J., Xiong Y., Bradley N., Wang D., DuBois R.N. (2020). Prostaglandin E2 Induces miR675-5p to Promote Colorectal Tumor Metastasis via Modulation of p53 Expression. Gastroenterology.

[96] Zhang L.-j., Chen B., Zhang J.-j., Li J., Yang Q., Zhong Q.-s., Zhan S., Liu H., Cai C. (2017). Serum polyunsaturated fatty acid metabolites as useful tool for screening potential biomarker of colorectal cancer. Prostaglandins Leukot. Essent. Fat. Acids.

[97] Saliakoura M., Reynoso-Moreno I., Pozzato C., Rossi Sebastiano M., Galie M., Gertsch J., Konstantinidou G. (2020). The ACSL3-LPIAT1 signaling drives prostaglandin synthesis in non-small cell lung cancer. Oncogene.

[98] Saul M.J., Baumann I., Bruno A., Emmerich A.C., Wellstein J., Ottinger S.M., Contursi A., Dovizio M., Donnini S., Tacconelli S. (2019). miR-574-5p as RNA decoy for CUGBP1 stimulates human lung tumor growth by mPGES-1 induction. FASEB J..

[99] Arima K., Ohmuraya M., Miyake K., Koiwa M., Uchihara T., Izumi D., Gao F., Yonemura A., Bu L., Okabe H. (2019). Inhibition of 15-PGDH causes Kras-driven tumor expansion through prostaglandin E2-ALDH1 signaling in the pancreas. Oncogene.

[100] Arima K., Komohara Y., Bu L., Tsukamoto M., Itoyama R., Miyake K., Uchihara T., Ogata Y., Nakagawa S., Okabe H. (2018). Downregulation of 15-hydroxyprostaglandin dehydrogenase by interleukin-1β from activated macrophages leads to poor prognosis in pancreatic cancer. Cancer Sci..

[101] Che D., Zhang S., Jing Z., Shang L., Jin S., Liu F., Shen J., Li Y., Hu J., Meng Q. (2017). Macrophages induce EMT to promote invasion of lung cancer cells through the IL-6-mediated COX-2/PGE2/β-catenin signalling pathway. Mol. Immunol..

[102] Wang J., Zhang L., Kang D., Yang D., Tang Y. (2018). Activation of PGE2/EP2 and PGE2/EP4 signaling pathways positively regulate the level of PD-1 in infiltrating CD8(+) T cells in patients with lung cancer. Oncol. Lett..

[103] Bergqvist F., Ossipova E., Idborg H., Raouf J., Checa A., Englund K., Englund P., Khoonsari P.E., Kultima K., Wheelock C.E. (2019). Inhibition of mPGES-1 or COX-2 Results in Different Proteomic and Lipidomic Profiles in A549 Lung Cancer Cells. Front. Pharmacol..

[104] Zong L., Li J., Chen X., Chen K., Li W., Li X., Zhang L., Duan W., Lei J., Xu Q. (2016). Lipoxin A4 Attenuates Cell Invasion by Inhibiting ROS/ERK/MMP Pathway in Pancreatic Cancer. Oxidative Med. Cell. Longev..

[105] Zong L., Chen K., Jiang Z., Chen X., Sun L., Ma J., Zhou C., Xu Q., Duan W., Han L. (2017). Lipoxin A4 reverses mesenchymal phenotypes to attenuate invasion and metastasis via the inhibition of autocrine TGF-β1 signaling in pancreatic cancer. J. Exp. Clin. Cancer Res..

[106] Wang Z., Cheng Q., Tang K., Sun Y., Zhang K., Zhang Y., Luo S., Zhang H., Ye D., Huang B. (2015). Lipid mediator lipoxin A4 inhibits tumor growth by targeting IL-10-producing regulatory B (Breg) cells. Cancer Lett..

[107] Fiala M., Halder R., Almasi A., Sagong B., Leung J., Jewett A. (2015). Curcuminoids and ω-3 fatty acids with anti-oxidants potentiate cytotoxicity of natural killer cells against pancreatic ductal adenocarcinoma cells and inhibit interferon γ production. Front. Physiol..

[108] Lee H.J., Park M.K., Lee E.J., Lee C.H. (2013). Resolvin D1 inhibits TGF-β1-induced epithelial mesenchymal transition of A549 lung cancer cells via lipoxin A4 receptor/formyl peptide receptor 2 and GPR32. Int. J. Biochem. Cell Biol..

[109] Sun Y.P., Oh S.F., Uddin J., Yang R., Gotlinger K., Campbell E., Colgan S.P., Petasis N.A., Serhan C.N. (2007). Resolvin D1 and its aspirin-triggered 17R epimer. Stereochemical assignments, anti-inflammatory properties, and enzymatic inactivation. J. Biol. Chem..

[110] Takano T., Fiore S., Maddox J.F., Brady H.R., Petasis N.A., Serhan C.N. (1997). Aspirin-triggered 15-epi-lipoxin A4 (LXA4) and LXA4 stable analogues are potent inhibitors of acute inflammation: Evidence for anti-inflammatory receptors. J. Exp. Med..

[111] Gilligan M.M., Gartung A., Sulciner M.L., Norris P.C., Sukhatme V.P., Bielenberg D.R., Huang S., Kieran M.W., Serhan C.N., Panigrahy D. (2019). Aspirin-triggered proresolving mediators stimulate resolution in cancer. Proc. Natl. Acad. Sci. USA.

